# Project IPAD, a database to catalogue the analysis of Fukushima Daiichi accident fragmental release material

**DOI:** 10.1038/s41597-020-00626-8

**Published:** 2020-08-28

**Authors:** Peter Martin, Omran Alhaddad, Yannick Verbelen, Yukihiko Satou, Yasuhito Igarashi, Thomas B. Scott

**Affiliations:** 1 School of Physics, University of Bristol, Tyndall Avenue, Bristol, BS8 1TL UK; 2 School of Computer Science, University of Bristol, Merchant Venturers Building, Bristol, BS8 1UB UK; 3 Collaborative Laboratories for Advanced Decommissioning Science, Japan Atomic Energy Agency, Tomioka-Machi, Futaba-gun, Fukushima 979-1151 Japan; 4 Institute for Integrated Radiation and Nuclear Science, Kyoto University, Kumatori-cho, Sennan-gun, Osaka 590-0494 Japan

**Keywords:** Nuclear waste, Environmental impact, Databases

## Abstract

The 2011 accident at Japan’s Fukushima Daiichi Nuclear Power Plant released a considerable inventory of radioactive material into the local and global environments. While the vast majority of this contamination was in the form of gaseous and aerosol species, of which a large component was distributed out over the neighbouring Pacific Ocean (where it was subsequently deposited), a substantial portion of the radioactive release was in particulate form and was deposited across Fukushima Prefecture. To provide an underpinning understanding of the dynamics of this catastrophic accident, alongside assisting in the off-site remediation and eventual reactor decommissioning activities, the ‘International Particle Analysis Database’, or ‘IPAD’, was established to serve as an interactive repository for the continually expanding analysis dataset of the sub-mm ejecta particulate. In addition to a fully interrogatable database of analysis results for registered users (exploiting multiple search methods), the database also comprises an open-access front-end for members of the public to engage with the multi-national analysis activities by exploring a streamlined version of the data.

## Abstract (summary)

**Table:** 

Measurement(s)	activity (of a radionuclide) • composition • isotopic ratio
Technology Type(s)	radioactivity measurement method • gamma-ray spectroscopy • transmission electron microscopy with EDAX • X-ray diffraction • micro-computed tomography • X-ray fluorescence microscopy • X-ray absorption spectroscopy • Raman spectroscopy • proton-induced X-ray emission spectroscopy • alpha-particle spectroscopy • beta-particle spectroscopy • secondary ion mass spectrometry • inductively coupled plasma mass spectrometry • thermal ionisation mass spectrometry • three dimensional-atom probe tomography • resonance ionisation mass spectrometry
Sample Characteristic - Environment	nuclear power plant
Sample Characteristic - Location	Fukushima Prefecture

Machine-accessible metadata file describing the reported data: 10.6084/m9.figshare.12821081

## Background & Summary

On the 11th March 2011, a 15 m high tsunami triggered by the Magnitude (M_W_) 9.0 Great Tõhoku Earthquake inundated Japan’s coastal Fukushima Daiichi Nuclear Power Plant (FDNPP)^1^. Despite the best efforts of the plants operators, [the] Tokyo Electric Power Company (TEPCO), to initiate appropriate core-cooling provision after it was destroyed by the tsunami^2^, the temperatures within each of the formerly operational reactor cores continued to rise over the succeeding days and weeks^3^. In the absence of adequate residual heat removal, there resulted a loss of coolant accident (LOCA) and the subsequent partial/complete meltdown of boiling water reactor (BWR) Units 1, 2 and 3 (alongside associated damage to the spent fuel storage pool of the neighbouring reactor Unit 4 resulting from the flow of flammable gases between shared ducting). Such integrity compromises of core, reactor pressure vessel (RPV) and primary containment vessel (PCV) resulted in a number of highly radioactive releases from the three reactor buildings – of which those from reactor Units 1 and 3 were violently explosive.

Of the estimated 520 PBq of radioactivity (excluding noble gases) released from all reactor units during the FDNPP accident^4,5^, 80% was transported and deposited offshore into the Pacific Ocean – with the remainder falling on land as a consequence of prevailing wind conditions at the time (via a combination of dry and wet deposition)^6^. While this 520 PBq globally significant release equates to only approximately 10% of the total source-term emitted from Chernobyl^4^, the events at the FDNPP were similarly rated at Level 7 (the most severe) on the International Atomic Energy Agency (IAEA) International Nuclear Event Scale (INES) due to the multiple reactors that together contributed to the accident.

The most significant on-land contamination is a consequence of the non-explosive release from reactor Unit 2^7^. However, in contrast to the highly visual releases from reactor Units 1 and 3, this release is invoked to have resulted from the eventual integrity failure of the PCV following the progressive pressure build-up from the extensive volumes of fission product and hydrogen gases produced^2,3,8^. Whereas the landward extent of the reactor Units 1 and 3 (explosive) radiological releases are associated with discrete areas inside a 5 km radius west of the plant^7,9^, the plume from reactor Unit 2 in considerably more spatially extensive – having deposited contamination along a 60 km north-westerly trace across Fukushima Prefecture^10^. Alongside gaseous release components (namely noble gases and other highly volatile species), the physical form of the particulate contamination associated with both release dynamics (explosive vs. non-explosive/effusive) is also contrasting^11^. The material released from reactor Unit 2, termed ‘Type A’ by Satou *et al*.^12^, comprises what has colloquially become known as ‘Cs-balls’ – highly spherical radiocesium-containing silicate-based micro-particles, each of approximately 2 μm in diameter, a small number of which have been observed to contain U at their centres^13,14^. Owing to their small dimensions and aerodynamic shape facilitating airborne transportation, these particles have been detected at considerable distances from the plant – including at monitoring locations greater than 100 km from the release site^15^. In contrast, the material invoked to have been derived from Unit 1 (and also potentially Unit 3), termed ‘Type B’ by Satou *et al*.^12^, is considerably larger (>200 μm in diameter) and more varied in both form, internal structure and composition^11,16^. Consequently, it is this suite of particulate (fragmental) material, collected from localities close to the FDNPP site, that is of greater interest in understanding the explosive release dynamics, the current state of the reactor(s) and how to undertake planned decommissioning activities.

In addition, such coarse diameter (>100 µm) material is also of interest from a dosimetry and population (irradiation) hazard viewpoint – including its potential uptake, ingestion and external radiological exposure. The occurrence of these sub-mm gamma-emitting particulates, which have been shown in earlier works to be resistant to surface weathering and erosional processes^16,17^, have the potential to represent a greater sustained internal and external radiation hazard than the volatilised (gaseous) radiocesium emitted and subsequently sorbed onto soil and mineral surfaces^18,19^.

**Fig. 1:**
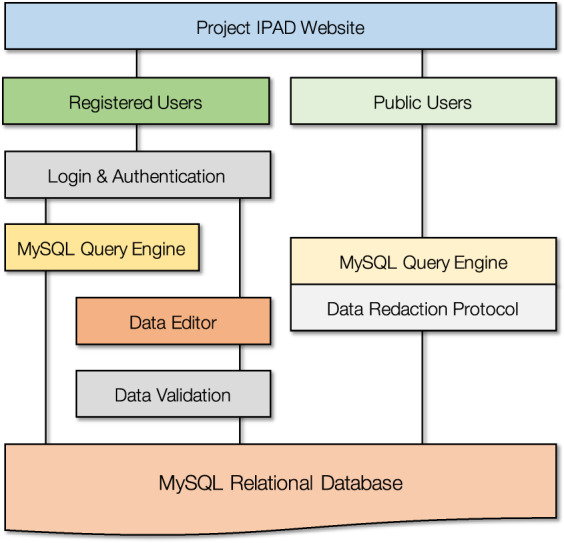
Representation of the database access, validation and query engine for both registered (academic) and public users. From the website ‘front-end’, approved users’ login to the relational database and are able to access the full ‘Data Editor’ and search functionality (MySQL Query Engine) within IPAD, whereas unregistered users (guests) are able to search only a reduced dataset (with a ‘Data Redaction Protocol’ being applied).

Therefore, to underpin the current international effort in (i) understanding the accident dynamics, and (ii) facilitating safe and efficient ‘fuel retrieval’ and decommissioning activities, the International Particle Analysis Database (IPAD) has been established. Whereas smaller (institutional or laboratory scale) databases of isolated particulate material and their subsequent analysis results exist, there occurs no centralised repository for collaborative applications to be based. Through the establishment and widespread support of this cross-institutional platform, it is envisaged that an enhanced understanding of the FDNPP accident and its decommissioning legacy can be ascertained – enhancing operational efficiency and safety through better underpinning science of the March 2011 accident. Public accessibility to all of the data deemed ‘non-sensitive’ (containing U and Pu isotopic information) is important in order to afford interested citizens with information on the accident and as a powerful scientific engagement tool – a schematic of this implementation as part of the database and reporting software architecture is shown in Fig. 1.

## Methods

The data contained within the Project IPAD database comprises searchable metadata and results from a wide range of analytical techniques, performed on particulate samples via a global network of collaborating laboratories and scientific facilities. Alongside the growing volume of particulate data comprising the database, serving to provide crucial information on the multiple reactor accident and potential decommissioning strategies, the architecture of the database itself continues to evolve to facilitate enhanced data searchability with greater filtering and refinement, in addition to including fields to document the results arising for new experimental techniques. The original Project IPAD included researchers from; (1) University of Bristol, (2) Japan Atomic Energy Agency, (3) Kyoto University, (4) Ibaraki University, (5) Osaka University, (6) University of Sheffield, (7) University of Tsukuba (8) Keio University, and (9) Tokyo University of Science. However, access to the database has subsequently been made available to all other interested/involved academic institutions and research organisations. The project and database implementation/delivery (to the necessary security, regulatory and data management requirements) also benefited from the support and collaboration of the University of Bristol Cabot Institute, UK Science and Technology Facilities Council (STFC), Engineering and Physical Sciences Research Council (EPSRC) and Amazon Web Services (AWS). The above partners and organisations brought to the project the necessary knowledge required to deliver a high-quality database, with the research institutions contributing their existing particle and analysis results databases.

### Data collection

The differing methodologies utilised to identify, isolate and extract the sub-mm radioactive particulate(s) from their containing matrix as well as subsequently obtain analysis results on such microscopic material using the wide range of experimental techniques is described in the extensive literature associated with the various works and studies^11,20^. These publications are listed in their entirety on the Project IPAD website. As subsequently discussed, outputs associated with/resulting from the analysis of a specific sample are additionally linked to that particle (via DOI and/or URL) using its enhanced metadata. The inventory of literature sources that underpins the results contained within the database currently stands at over 140 publications – each analysing an average of 5 particulate samples. Such works utilise a complimentary combination of both non-destructive and destructive analysis techniques^21,22^. Resulting from the large number of multi-national organisations, laboratories and facilities contributing to the portfolio of analysis results; a range of instruments, experimental setups and data output formats are associated/produced.

#### Non-Destructive measurements

These analysis methods typically comprise the first techniques to be undertaken and serve to provide initial information on the particulate material – namely its radioactivity, form/structure and elemental composition, all without any damage to the sample or removal of material. This is achieved through conventional materials science and radiation counting techniques, which are foundational techniques of such characterisation laboratories worldwide.

Following the particulates isolation from the bulk (e.g. soil, aerosol filter, dust), the first methodology applied to the sample is gamma-ray (γ) spectroscopy. Through the detection and quantification of the gamma-ray photons emitted by the sample, typically using a shaped crystal of cryogenically cooled high-purity germanium (HPGe), the contributing radionuclides can be determined alongside their relative abundances^23^. For such Fukushima-derived material, the decay-corrected ^134^Cs/^137^Cs activity ratio has been shown to represent a crucial indicator of the materials specific reactor provenance^12^, following modelling of the differing core burn-up scenarios^24^.

After determining if the decay-corrected (to March 2011) activity ratio of ^134^Cs/^137^Cs is either <1 (Unit 1) or >1 (Unit 2 or Unit 3) and therefore the particles likely emission source (most particulates >10 μm and contained within IPAD are attributed to have been released from reactor Unit 1), subsequent non-destructive testing is performed within the scanning electron microscope (SEM). Typically equipped with energy dispersive spectroscopy (EDS) detectors, the SEM (using various integrated detector options) is capable of producing images of the surface of particulate samples at nm spatial resolution – with EDS affording complimentary surface compositional characterisation at 0.1 wt% levels of detection^25^.

While γ-ray spectroscopy, SEM and EDS together constitute the primary non-destructive characterisation methods, further techniques are also applied to such sub-mm fallout particulate – with their results similarly contained within the IPAD platform. In contrast to SEM and EDS analysis, which utilise a highly focused beam of electrons to examine a material, x-rays can also be used to study a sample. Whether employing laboratory or ‘brighter’ and more intense synchrotron x-ray sources, such x-ray techniques include; x-ray diffraction (XRD) – to determine the constituent phase chemistry; x-ray tomography (XRT) – obtaining a series of absorption contrast images which when combined produce a 3D reconstruction of the particle; x-ray fluorescence (XRF) – examining the characteristic x-ray energies emitted to elucidate whole particle or point elemental composition and x-ray absorption spectroscopy (XAS) to derive co-ordination chemistry, oxidation states and bonding (including x-ray absorption near edge structure, XANES, and extended x-ray absorption fine structure, EXAFS)^21,22^.

Further non-destructive techniques applied to such fine scale, yet highly radioactive, particles include Raman spectroscopy for compositional analysis, and proton-induced x-ray emission (PIXE), an additional form of spectroscopy using an incident proton beam (rather than x-rays or electrons) to induce an x-ray emission (through a mechanism analogous to XRF and EDS) via which to study the particles composition. Less common non-destructive techniques that have been surpassed by modern alternatives include both alpha (α) particle spectroscopy and the similar beta (β) particle spectroscopy. Similar to γ-ray spectroscopy, both methodologies also examine and quantify the specific energy of the emitted radiation, albeit in this instance the subatomic α and β particles, to determine the radioisotope from which it was emitted as well as the associated specific activity.

#### Destructive measurements

In support of the aforementioned non-destructive analysis techniques, a number of additional methodologies that require the consumption of some, or all, of the sample exist. Unlike the formerly described methods of sample characterisation, such destructive techniques typically afford highly accurate compositional and/or isotopic information on the material, utilising mass spectrometry methods. A mainstay of high-accuracy (and spatial) isotopic analysis is secondary ion mass spectrometry (SIMS), whereby ejecta species produced following a samples ablation and fragmentation with a primary ion beam are isotopically analysed for their mass/charge ratio^26^. Additional techniques applied to quantify the isotopic composition of such samples include; inductively coupled plasma – mass spectrometry (ICP-MS) and thermal ionisation mass spectrometry (TIMS) – whereby prior sample preparation permits for their injection into an Ar carrier plasma for similar high-precision atomic analysis^27,28^.

These more mainstream and widely adopted techniques are supported by an increasing number of more novel methodologies to quantify a particulates isotopic composition. Three dimensional-atom probe tomography (3D-APT) is one such approach^29^, as is resonance ionisation mass spectrometry (RIMS)^30^ – both of which utilise extremely small volumes of sample material (<10^7^ atoms) while affording a low limit of detection as well as atomic-scale isotopic mapping in the case of 3D-APT. A further technique that requires the destructive analysis of a (small) portion of the sample as part of the preparation phase is transmission electron microscopy (TEM). In a process similar to creating an ultra-fine 3D-APT filament, a focused ion beam (FIB) instrument is used to produce a thin (<100 nm) ‘foil’ of material through which a high energy beam of electrons can pass. While unable to derive isotopic information on the sub-sampled portion of the particle, TEM is capable of obtaining nm-resolution compositional/phase information in addition to crystallographic and structural data on the thin sample slice.

## Data Records

The datasets are available at Martin, P. Project IPAD, a database to catalogue the analysis of Fukushima Daiichi accident fragmental release material. Mendeley 10.17632/nz6hjbfs65.3 (2020)^31^. The associated Project IPAD website (www.projectipad.org) offers both academic (registered) and public users’ access to queries and reports performed on the underlying MySQL Relational Database. These are based upon search parameters as defined by the user. These reports are downloadable in both.html and.pdf formats and can be configured to include user-specified data fields. Registered users are able to run queries on the full Relational Database via a MySQL Query Engine. Public reports derived from the database are possible provided that they do not contain reference to the small volume of the data that comprises U and Pu isotopic results – with this filtering performed through a ‘Data Redaction Protocol’ associated with the Public MySQL Query Engine – as shown in the schematic of Fig. 1.

**Fig. 2:**
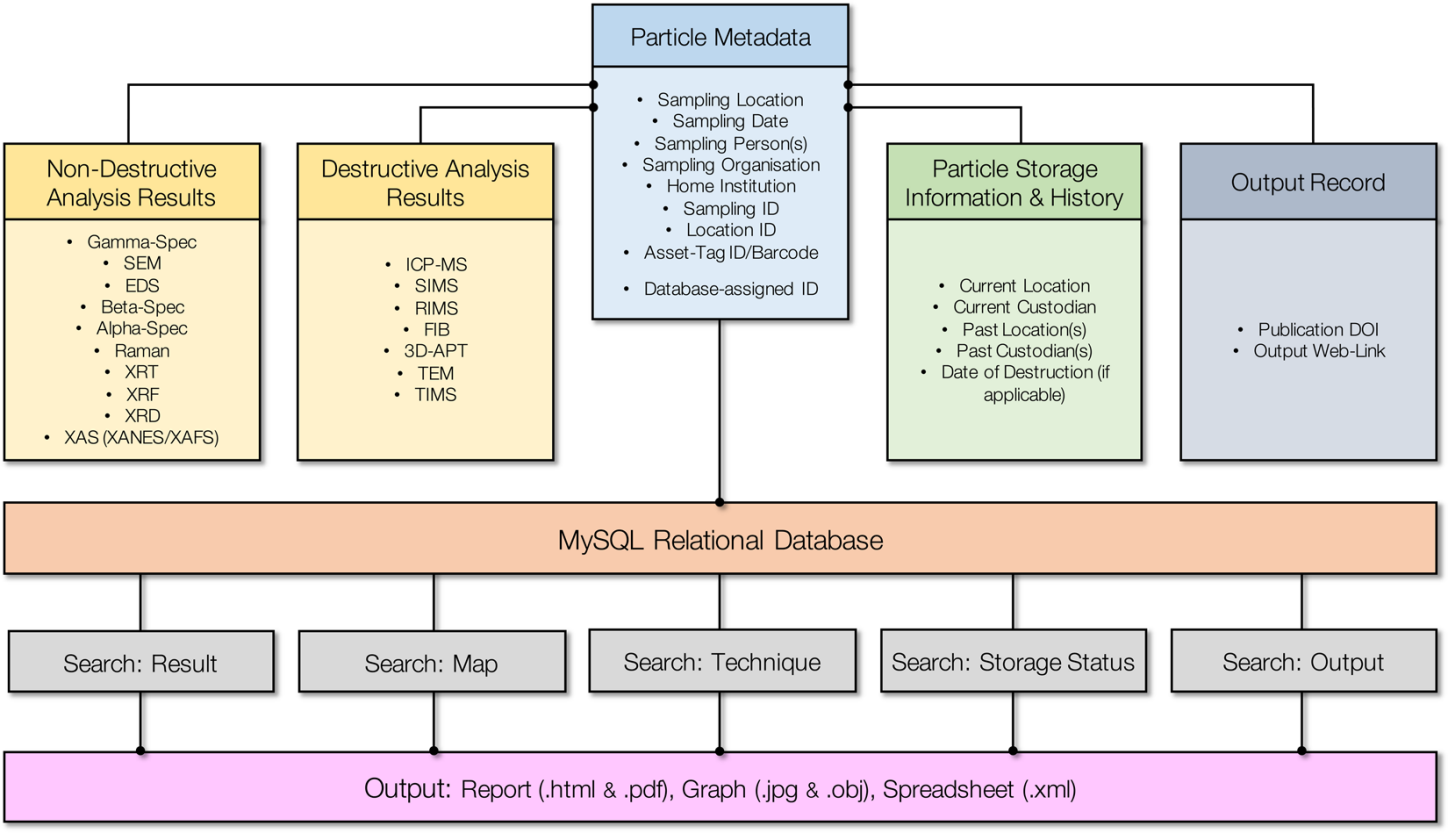
Structure of Project IPAD, comprising; analysis results (yellow), storage information/history (green) and publication details (grey) all attached onto the particle metadata (blue) which is contained within the searchable (via various methods) relational database to yield output reports and spreadsheets.

All of the raw data contained within the relational database resulting from the suite of analytical techniques is stored ‘linked’ to each individual particle and its associated searchable metadata. This metadata (including fields such as; date of collection, sampling location, sampling institution and material custodian) is input by the user prior to such analysis results being uploaded and associated with the sample. A schematic of this relationship is shown in Fig. 2. Alongside the ‘primary’ particle metadata (blue in Fig. 2) and analysis result datasets (shown in yellow), additional metadata is associated with each particle record. This ‘secondary’ metadata comprises; (i) the storage, custody and management of the radioactive sample material (shown in green) as well as a record of the published works and outputs resulting from the analysis of a particular particle sample (shown in grey). Further metadata (in addition to the aforementioned ‘primary’ particle information) is generated alongside the upload of raw analysis results. Such user-input metadata fields are also searchable via MySQL queries of the relational database, whereas the unprocessed data files that are uploaded serve only as repository material if users wish to subsequently download, examine and reprocess results. The IPAD platform permits for multiple analysis results using the same technique on the same sample to be uploaded and differentiated by the user.

To afford database users with an easily discernible view of what analysis techniques have been undertaken on what samples, therefore removing the need to probe the database for the relevant data, indicator icons are presented alongside each samples metadata entry on the results landing/home page. Through these icons, users can directly access the applicable raw datasets without entering each particles individual metadata-tethered record. The raw datasets are downloadable in each instance in their native upload format. Users who upload experimental data onto the IPAD system are also encouraged to additionally upload small explanatory files describing any processing that has been performed on the data, or any such work that should be undertaken subsequently by a user.

As a primary goal of Project IPAD is the dissemination and collaborative analysis of large quantities of Fukushima-derived particulate data, the ability of users to download and process such experimental results as part of their own analysis is key. To facilitate this, the datasets uploaded to the database are in standard and widely accepted/adopted formats for that analysis method. A summary of the analytical techniques, associated data (file) formats is detailed in Table 1. While it is expected that users accessing particle data associated with a specific experimental technique (for example, synchrotron XAS) will be familiar with the associated file formats that the results are customarily presented in (e.g. .hdf5 and .nxs), information on all file formats and links to the relevant software downloads are contained within the IPAD system. However, while it may not be possible for some analysis types or when specific equipment is used for data collection, where options exist for the data to be in ‘standard’ formats (e.g. .txt, .csv, .spc, .html, .tiff, .jpg and .msa) that does not necessitate users download additional software, a strong preference is expressed at the data upload stage.

**Table 1:** Summary of the analytical techniques and associated data (file) formats contained within the IPAD system (* the .doc format is used to export a series of XRT images from the instrument within a predefined .doc template as a means to capture representative results with a reduced total file size, rather than the entire XRT dataset/stack).

Technique Category	Analysis Technique	Standard Output File Format
Non-Destructive	Gamma-Ray Spectroscopy (γ-spec)	.txt
	Scanning Electron Microscope (SEM) Imaging	.jpg .tiff
	Energy Dispersive Spectroscopy (EDS)	.csv .spc .msa
	Raman Spectroscopy (RS)	.csv
	X-Ray Diffraction (XRD)	.csv
	X-Ray Tomography (XRT)	.tiff .txm .jpg (.doc*)
	Alpha-Spectroscopy (α-spec)	.spc .csv .txt
	Beta-Spectroscopy (β-spec)	.spc .csv .txt
	X-Ray Fluorescence (XRF)	.spc .csv .txt
	Proton Induced X-Ray Emission (PIXE) Spectroscopy	.nxs .csv .txt .hdf5
	X-Ray Absorption Spectroscopy (XAS)	.nxs .csv .txt .hdf5
Destructive	Secondary Ion Mass Spectrometry (SIMS)	.csv .txt
	Inductively Couple Plasma - Mass Spectrometry (ICP-MS)	.csv .txt
	Thermal Ionisation Mass Spectrometry (TIMS)	.csv .txt
	Resonance Ionisation Mass Spectrometry (RIMS)	.csv .txt
	Three Dimensional-Atom Probe Tomography (3D-APT)	.HITS
	Transmission Electron Microscopy (TEM)	.jpg .tiff .csv .txt

The suite of analytical techniques listed within the IPAD system represents the current portfolio of state-of-the-art and widely adopted characterisation methodologies for radioactive micro-particle analysis found at laboratories worldwide. These techniques are capable of both destructive and non-destructive qualitative and quantitative analysis at varying resolutions or detection limits – a summary of the attainable/accepted analysis limits are shown in Table 2. It is, however, anticipated that with technique advancement and evolution that the IPAD database will subsequently evolve to capture results captured through other analytical methods.

**Table 2:** Summary of the currently attainable measurement resolutions and limits of detection (LoD) for the analytical methodologies presently listed within the IPAD system.

Technique Category	Analysis Technique	Measurement Property	Typical Measurement Resolution /Limit of Detection (LoD)
Non-Destructive	γ-spec	Isotopic (γ-emitting) Composition	>0.01 Bq/g (γ)
	SEM	2D Imaging	1 nm
	EDS	Elemental Composition	>0.1 wt%
	RS	Phase Composition	parts per thousand *(N.B. strongly material dependent)*
	XRD	Phase Composition	parts per million
	XRT	3D Imaging	100 nm – 1 µm
	α-spec	Isotopic (α-emitting) Composition	>0.01 Bq/g (α)
	β-spec	Isotopic (β-emitting) Composition	>0.01 Bq/g (β)
	XRF	Elemental Composition	>0.1 wt%
	PIXE	Elemental Composition	parts per million
	XAS	Elemental and Phase Compositions Oxidation State Chemistry & Coordination	parts per million
Destructive	SIMS	Isotopic Composition	parts per billion
	ICP-MS	Isotopic Composition	parts per billion
	TIMS	Isotopic Composition	parts per billion
	RIMS	Isotopic Composition	parts per billion
	3D-APT	Isotopic Composition	parts per billion
	TEM	2D Imaging Elemental and Phase Composition	<1 nm <parts per million

**Fig. 3:**
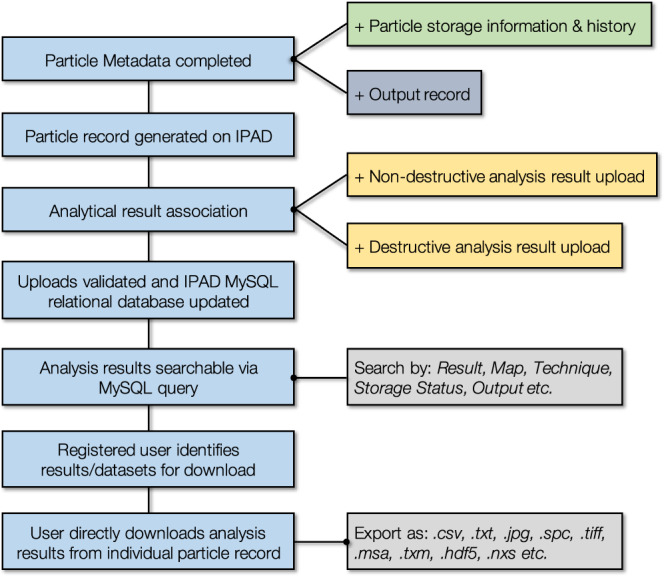
Multistep workflow for the user generation of a particle record, association of metadata, upload of analytical results, multi-parameter search of results and subsequent download of result dataset.

As shown in Fig. 2, the database can be interrogated for results and experimental data (for download and subsequent processing) through a number of search mechanisms – a schematic of the process workflow is shown in Fig. 3. These search queries include any one or a combination of; (i) result specific [i.e. samples with a ^134^Cs/^137^Cs activity ratio of >1.0], (ii) technique applicable [e.g. whether a certain analytical methodology has been applied], (iii) map search [graphically selecting data from particulate material obtained from a user-specified region], (iv) storage status/location [the location, home institution or whether the material has been destroyed as part of analysis activities, and (v) applicable publication outputs [if sample analysis results have contributed to or featured within published works].

## Technical Validation

Prior to the Project IPAD platform being made available live online, it was thoroughly tested and verified. A comprehensive inventory of simulated material (comprising metadata and experiment results) was uploaded to the database; with all aspects of the functionality of the system evaluated. Supported by regular (every 30 minute) backup snapshots of the data contained within the database, the design of the system is such that the full data record is independent of the website and query engines that interface into it. A full edit history/log is also maintained for each database entry, where it is possible to review each edit step, reverting any such changes made.

To ensure data integrity is maintained throughout Project IPAD, in terms of both the user-input metadata and the associated experimental results/raw data, multiple validation processes are integrated within the MySQL database structure. During the manual input of data and summary results into the text fields of the web based IPAD interface, there exists the significant potential for transcription errors to arise, as well as variations in naming/convention (i.e. sampling locality, name of person/collector and institution) owing to differences in English/Japanese spelling and/or word-order.

In order to explicitly combat these, the number of ‘free text’ fields within the database is limited – replaced with dropdown lists of predefined values in instances where the total number of options is not likely to increase extensively and therefore require regular modification to the underlying database to list these new possibilities. However, where new options/selections are likely to often arise, suggestive inputs (based upon results formerly entered into the database) are shown as typing commences. In addition to eliminating such textual issues that would necessitate subsequent data amendment to ensure the full searchability of the particulate inventory, completeness of the data entry is mandated by users not being able to continue with the workflow unless all input fields have been completed. Alongside eliminating such naming inconsistencies, a form of input validation (against specified parameters) is associated with each field to ensure that it conforms with data quality requirements. A summary of these automatic validation conditions is presented in Table 3. Also shown in Table 3 are the validation requirements of the uploaded (experimental) datasets.

**Table 3:** Data validation requirements applied to both user-input data/results and experimental datasets uploaded onto the Project IPAD platform.

	Data Field	Validation Requirement
Particle Metadata (User-Input)	Sample Location	From list
	Sample Location Reference	letter.letter-number.number
	Sample ID	Number of characters <10
	Sample Name	Number of characters <10
	Date of Collection	dd/mm/yyyy (must be in past)
	Sampling Location (Lat. and Long.)	As: xx.xxxxxx – yy.yyyyyy
	Sample Collector	From list
	Sampling Institution	From list
	Home Location	From list
	Sample Status	From list
	Publication DOI	As: 10.xxxx/xxxxx
	Asset Tag ID	All numerical digits
Experimental Results	Percentages	Sum to 100%
	^134^Cs/^137^Cs Activity Ratio	0.9–1.2, to include errors
	All .csv files	Contain ≥2 columns
	All .jpg and .tiff files	<25 Mb per file size
	All .txt files	<50 Mb per file size
	Isotope Ratio(s)	Within defined range, to include errors

## Data Availability

All data records were generated using code developed in MySQL, using the Laravel Framework. The database source code is available upon request from the corresponding author.
